# Canine leishmaniasis: Genome-wide analysis and antibody response to *Lutzomyia longipalpis* saliva

**DOI:** 10.1371/journal.pone.0197215

**Published:** 2018-05-09

**Authors:** Luís F. S. Batista, Yuri T. Utsunomiya, Thaís B. F. Silva, Mariana M. Carneiro, Joyr S. F. Paiva, Rafaela B. Silva, Thaíse Y. Tomokane, Claudio N. Rossi, Acácio D. Pacheco, Rafaela B. P. Torrecilha, Fernando T. Silveira, Mary Marcondes, Cáris M. Nunes, Márcia D. Laurenti

**Affiliations:** 1 Departamento de Patologia Veterinária, Faculdade de Medicina Veterinária e Zootecnia, Universidade de São Paulo, São Paulo, Brazil; 2 Escola de Saúde, Universidade Salvador, Salvador, Bahia, Brazil; 3 Departamento de Medicina Veterinária Preventiva e Reprodução Animal, Faculdade de Ciências Agrárias e Veterinárias, Univ Estadual Paulista, Jaboticabal, São Paulo, Brazil; 4 Laboratório de Patologia de Doenças Infecciosas, Faculdade de Medicina, Universidade de São Paulo, São Paulo, Brazil; 5 Departmento de Clínica, Faculdade de Medicina Veterinária e Zootecnia, Universidade de São Paulo, São Paulo, Brazil; 6 Departamento de Clínica, Cirurgia e Reprodução Animal, Faculdade de Medicina Veterinária, Univ Estadual Paulista, Araçatuba, São Paulo, Brazil; 7 Deparatmento de Parasitologia, Instituto Evandro Chagas, Ananindeua, Pará, Brazil; 8 Departmento de Saúde Animal e Produção, Faculdade de Medicina Veterinária, Univ Estadual Paulista, Araçatuba, São Paulo, Brazil; University of Ostrava, CZECH REPUBLIC

## Abstract

The anti-inflammatory properties of sand fly saliva favor the establishment of the *Leishmania infantum* infection. In contrast, an antibody response against *Lutzomyia longipalpis* saliva is often associated with a protective cell-mediated response against canine visceral leishmaniasis. Genetic studies may demonstrate to what extent the ability to secrete anti-saliva antibodies depends on genetic or environmental factors. However, the genetic basis of canine antibody response against sand fly saliva has not been assessed. The aim of this study was to identify chromosomal regions associated with the anti-*Lu*. *longipalpis* salivary IgG response in 189 dogs resident in endemic areas in order to provide information for prophylactic strategies. Dogs were classified into five groups based on serological and parasitological diagnosis and clinical evaluation. Anti-salivary gland homogenate (SGH) IgG levels were assessed by Enzyme-Linked Immunosorbent Assay (ELISA). Genomic DNA was isolated from blood samples and genotyped using a SNP chip with 173,662 single nucleotide polymorphism (SNP) markers. The following linear regression model was fitted: IgG level = mean + origin + sex + age + use of a repellent collar, and the residuals were assumed as pseudo-phenotypes for the association test between phenotypes and genotypes (GWA). A component of variance model that takes into account polygenic and sample structure effects (EMMAX) was employed for GWA. Phenotypic findings indicated that anti-SGH IgG levels remained higher in exposed and subclinically infected dogs than in severely diseased dogs even in regression model residuals. Five associated markers were identified on chromosomes 2, 20 and 31. The mapped genes included *CD180* (*RP105*) and *MITF* related to the rapid activation of B lymphocytes and differentiation into antibody-secreting plasma cells. The findings pointed to chromosomal segments useful for functional confirmation studies and a search for adjuvant molecules of the anti-saliva response.

## Introduction

*Leishmania infantum* (synonymous with *Leishmania chagasi*) infection ranges from asymptomatic infection to fatal visceral leishmaniasis (VL) in up to 10% of treated patients [1]. Brazil, where VL is zoonotic, contributes 94% of cases reported in the Americas and has the third position in worldwide VL prevalence [2, 3]. The domestic dog (*Canis lupus familiaris*) is the main urban reservoir of *L*. *infantum*. Dogs are a source of infection for the female *Lutzomyia longipalpis* sand fly, the major vector of VL in the Americas. Therefore, dogs have a significant role in the transmission of zoonotic VL [4].

The coevolutionary adaptation of sandflies and *L*. *infantum* includes the contribution of a repertoire of molecules present in sand fly saliva [5]. Salivary gland homogenate (SGH) affects the host hemostatic, inflammatory and immunological responses. Thus, sand fly saliva facilitates the blood meal needed for egg maturation [5]. Many of the pharmacologically active molecules present in saliva have been reported to be immunogenic based on induction of a host immune response [6,7]. After saliva deposition in the host dermis, recruitment of neutrophils, macrophages and lymphocytes occurs. These last trigger a delayed hypersensitivity response (DTH) at the bite site [7]. This cell-mediated response is extensively associated with an increased anti-saliva antibody response, mainly IgG2 in humans [8] and dogs [7]. The multiple effects of the sand fly saliva contribute to the establishment of *L*. *infantum* infection [9, 7]. In contrast, treatment with anti-SGH antibody or pre-exposure to the bite of *Lu*. *longipalpis* has been shown to reverse the saliva effects [9, 7]. This information along with the increase in anti-*Lu*. *longipalpis* SGH IgG levels in *L*. *infantum*-negative or subclinically infected dogs compared to diseased dogs [10, 11] supports the hypothesis that an antibody response to sand fly saliva plays a protective role in the outcome of canine *L*. *infantum* infection. In addition, the antibory response can be interpreted as an exposure sensor. Increased levels of anti-*Lu*. *longipalpis* SGH IgG, IgG1 and IgG2 were noted in animals more heavily exposed to the bite of *Lu*. *longipalpis*; however, this response was not common to all exposed dogs [7, 10]. Such a pattern of response suggests variability in the genetic basis of anti-saliva IgG secretion capacity.

The employment of a genome-wide association study (GWAS) may further contribute to the elucidation of processes inherent to responses to vectors. Therefore, the purpose of the present study was to identify chromosome segments sheltering genetic bases for the quantitative variations of anti-*Lu*. *longipalpis* saliva antibodies in the sera of dogs from endemic areas of VL, previously reported by our group [11]. Our findings indicated five chromosomal regions that may be useful for identifying new targets for functional and prophylactic explorations.

## Methods

### Sampling and sample structure

A multi-breed sample of dogs living in an endemic area was evaluated. Variables whose effects are potentially confounding in the expression of phenotypes, such as sex, age, geographic origin, and use of repellent collars, were included as fixed-effect covariates in the regression model. Residency of dogs for at least 18 months in 11 distinct endemic areas, distributed among the Brazilian states of Bahia, Minas Gerais, São Paulo and Federal District, and an absence of history of comorbid infectious diseases (epidemiological survey by questionnaire) were the inclusion criteria for the study. A sample size calculation was not performed due to a lack of previous studies demonstrating the size of the effect of genetic variables on the expression of anti-*Lu*. *longipalpis* saliva IgG levels. Therefore, the determination of the sample size was limited to the number of dogs whose owners granted participation and by the cost of the inputs for the genotyping step. The sample structure was evaluated by principal component analysis (PCA). A genetic relationship matrix among the sampled breeds was constructed based on the first two major components in the RStudio version 0.98.1103 program (available at: https://www.r-project.org/). This study was conducted according to the Ethical Principles in Animal Research adopted by the Brazilian College of Animal Experimentation and approved by the Ethics Committee in the use of animals of the School of Veterinary Medicine and Animal Science from the University of São Paulo under protocol 2391/2011.

### Parasitological diagnosis

Popliteal or pre-scapular lymph node fine needle aspiration biopsies (LN) and buccal (BS) and conjunctival (CS) swabs were collected, maintained in NET buffer (0.15 M NaCl, 50 mM EDTA, 0.1 M Tris HCl; pH 7.5) and stored at 4°C. DNA was isolated using a commercial kit (NucleoSpin® Tissue, Macherey Nagel, Germany), according to the manufacturer's instructions. Real-time polymerase chain reaction (PCR) was performed as described by Batista et al. [11] using primers targeting a 120 bp kDNA sequence of *L*. *infantum*. The specificity of the amplified sequence was assumed based on overlaying the melting curve of *L*. *infantum* DNA (MHOM/BR/72/strain 46) and the divergence from control groups of *Leishmania amazonensi*s (MHOM/BR/73/M2269) and *Leishmania braziliens*is (MHOM/BR/1995/M15280) [12, 13].

### Sand fly collection and preparation of salivary gland homogenate (SGH)

Wild-caught *L*. *longipalpis* sand flies were used. The wild-caught sand flies were collected in Barcarena municipality located in Para State, Brazil over a period of 3 days, although those caught in the first collection were not used in order to work with newly released sandflies of similar age. These sandflies were maintained in the insectary for two days until the time of the experiments. The wild-caught sandflies were checked for the absence of blood meal in their guts and egg development in their ovaries in order to confirm that they had no previous blood intake. All the sandflies received 5% sugar solution ad libitum until the time of the experiments. They were maintained in the insectary of the Laboratory of Leishmaniasis at Evandro Chagas Institute according to the conditions described by Killick-Kendrick et al. [14]. No sand flies were captured in a protected area, national park or private area, just as no protected or endangered species were involved in the study. Therefore, no specific permission was required.

Salivary gland lysates were obtained from wild-caught sandfly females. At the moment of the dissection, all the sandfly midguts were verified regarding the absence of blood meal. The dissected salivary glands were collected in phosphate buffered saline (PBS), pH 7.2, and stored at − 70°C. At the time of the experiments, the salivary glands were disrupted by freeze-thawing, vortex and rapid centrifugation to discharge possible tissues. The protein concentration was determined by Bradford.

### Quantification of anti-*L*. *infantum* and anti-sand fly saliva IgG

An indirect enzyme-linked immunosorbent assay (ELISA) using crude *L*. *infantum* antigen (MHOM/BR/72/strain 46) and anti-canine IgG (A40-123AP, Bethyl, USA) was performed according to Laurenti et al. [15] to evaluate the anti-*L*. *infantum* antibody level. The anti-sand fly SGH IgG level was evaluated by ELISA following Batista et al. [11].

### Clinical evaluation

Clinical signs consistent with CanL, such as dermatitis, alopecia, hyperkeratosis, onychogryphosis, lymphadenopathy, splenomegaly, hepatomegaly, emaciation, conjunctivitis, uveitis and blepharitis, were evaluated and recorded in a single visit to the property. The quantification of biochemical markers was performed using an automated spectrophotometer (BS 200, Shenzhen Mindray Bio-Medical Electronics Co., Nanshan, China) previously calibrated with serum control levels I and II (Biosystems, Barcelona, Spain). The biochemical markers and methods used for the determination of their concentrations were as follows: albumin (g/L) by the bromocresol green method; urea (mg/dL) by the urease/glutamate dehydrogenase assay coupled with the UV enzymatic method; creatinine (mg/dL) by the kinetic alkaline picrate assay; total plasma protein (g/L) by the biuret method; aspartate aminotransferase (AST, IU/L) and alanine aminotransferase (ALT, IU/L) by the enzymatic UV method following the International Federation of Clinical Chemistry (IFCC) guidelines; and alkaline phosphatase (IU/L) by the diethanolamine method. Globulin levels (g/L) were determined by the difference between total protein and albumin. Reference values for biochemical parameters were based on Kaneko et al. [16]. Dogs were ranked according to the adapted clinical staging according to Paltrinieri et al. [17] as follows: Group I–uninfected, PCR and serology negative dogs from endemic area; Group II–exposed, PCR negative dogs with low anti-*L*. *(L*.*) infantum* IgG level (up to twice the threshold value); Group III–infected, PCR positive, apparently healthy dogs; Group IV–diseased, PCR positive dogs presenting mild signs such as localized lymphadenopathy, dermatitis, alopecia, onychogryphosis and ocular damage; and Group V—severely diseased, PCR positive dogs presenting with systemic lymphadenopathy, splenomegaly, hepatomegaly, emaciation or altered mobility as well as signs of Group IV (**Table 1**).

**Table 1:** Proportion of positive results in the parasitological and serological diagnosis and clinical and biochemical findings of each clinical group.

Clinical group (n)	PCR+/tested	ELISA+/tested	Clinical findings/tested	Biochemistry findings/tested*
**I. Uninfected (21)**	0/21	0/21	0/21	Hypergammaglobulinemia (0/21) ALT (7/21) AST (6/21) Creatinine (4/21) Uremia (2/21)
**II. Exposed (12)**	0/12	12/12	0/12	Hypergammaglobulinemia (0/12) ALT (4/12) AST (3/12) Creatinine (2/12) Uremia (1/12)
**III. Infected (56)**	56/56	18/56	0/56	Hypergammaglobulinemia (10/56) ALT (9/56) AST (16/56) Creatinine (9/56) Uremia (9/56)
**IV. Diseased (52)**	52/52	39/52	Pale mucosa (5/52) Lymphadenomegaly (45/52) Alopecia (26/52) Dermatitis (27/52) Hyperkeratosis (13/52) Onychogryphosis (9/52) Ocular damage (13/52)	Hypergammaglobulinemia (35/52) ALT (29/52) AST (33/52) Creatinine (13/52) Uremia (12/52)
**V. Severely diseased (24)**	24/24	24/24	Pale mucosa (6/24) Lymphadenomegaly (19/24) Alopecia (16/24) Dermatitis (16/24) Hyperkeratosis (9/24) Onychogryphosis (15/24) Ocular damage (11/24) Systemic lymphadenomegaly (4/24) Splenomegaly (17/24) Hepatomegaly (7/24) Emaciation (3/24)	Hypergammaglobulinemia (21/24) ALT (14/24) AST (18/24) Creatinine (6/24) Uremia (5/24)

* Reference values according to Kaneko et al. [16]

### Statistical analysis of phenotypic data

The distribution of the quantitative values of anti-SGH IgG in ELISA units was evaluated by descriptive statistics parameters, such as the mean, standard deviation, coefficient of variance and kurtosis level, using R Studio software. The distribution was considered asymmetric when the standard deviation was greater than 50% of the mean. A nonparametric Kruskal-Wallis test was adopted to compare antibody levels with asymmetric distribution among Groups I to V in R Studio software v3.2.3 (available at: https://www.r-project.org/).

### Genotyping

DNA was isolated from whole blood samples with a commercial kit (NucleoSpin® Tissue, Macherey Nagel, Germany) and genotyped for 173,662 single nucleotide polymorphism (SNP) markers with the Illumina® CanineHD BeadChip assay (Illumina Inc., San Diego, CA, USA), according to the manufacturers' protocols.

### Genome wide association (GWA) analysis

Prior to the GWA analysis, the following linear model was fitted in R Studio v3.2.3: anti-*Lu*. *longipalpis* SGH IgG level _~_ mean + origin + sex + age + repellent collar.

For the analysis of antibody response (quantitative data), an ordinary least squares regression was used. Then, residuals of the fitted models were used as pseudo-phenotypes to test the association between genotypes and phenotypes in a mixed linear model controlled for polygenic effects and sample structure (EMMAX) [18] in SNP & Variation Suite (SVS) v.8 (Golden Helix, Inc., Bozeman, MT, USA, www.goldenhelix.com). Genotypes were filtered in order to remove SNPs with a call rate below 95%, a minor allele frequency (MAF) below 5% and a p-value in an exact test for Hardy-Weinberg equilibrium (HWE) less than 10^−5^. Samples with a call rate less than 90% were also removed. Markers were prioritized for investigation based on a significance level of p <1 × 10^−5^ [19]. Gene coordinates in the CanFam v3.1 assembly were obtained from Ensembl Genes 84 using the BioMart tool (available at: http://www.ensembl.org/biomart/martview/). Genes mapping to a maximum distance of 1 Mb from associated markers were considered positional candidates. The inflation factor and variance explained by the markers were obtained in SVS.

## Results

### Sampling and sample structure

**Fig 1:**
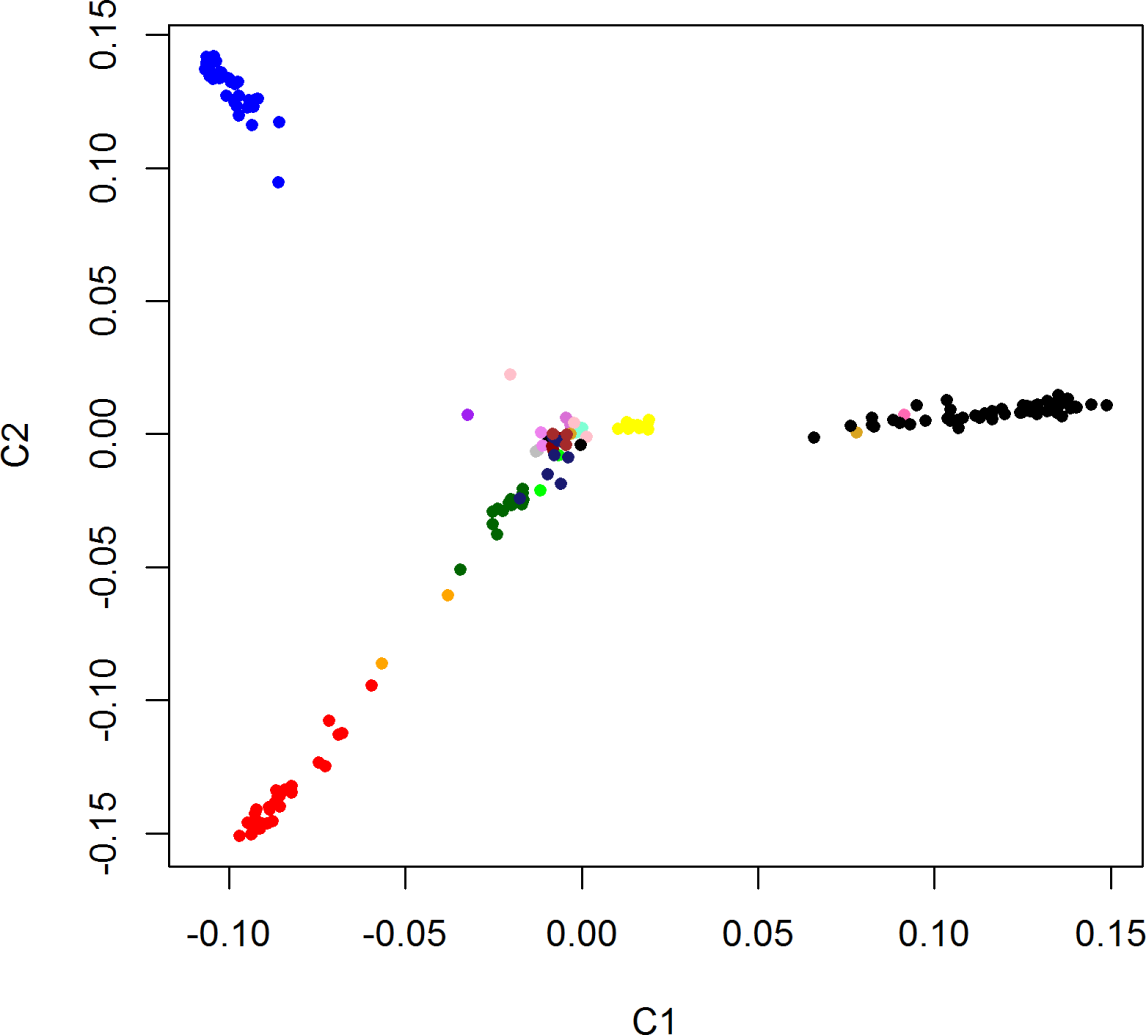
Principal component analysis (PCA) showing the genetic relationship between the sampled breeds and the grouping of the first two main components (C1 and C2) in three homogeneous sets and three heterogeneous sets. Labrador Retriever (black), Italian Greyhound (goldenrod), Cocker Spaniel (pink), Rottweiler (blue), German Shepherd Dog (forest green), Belgian Shepherd Dog (red), White Swiss Shepherd Dog (orange), and Golden Retriever (yellow).

The multi-breed sample that included 62 Labradors, 35 Rottweilers, 32 German Shepherds, 15 Belgian Shepherds, 10 Golden Retrievers, 3 Boxers, 2 Cocker Spaniels, 2 Dobermans, 2 White Swiss Shepherds, 2 Miniature Pinschers, 2 Saint Bernards, 2 German Spitzs, 1 Bloodhound, 1 Dogo Argentino, 1 Wire-haired Fox Terrier, 1 Italian Greyhound, 1 Poodle and 8 mixed-breeds was analyzed. These included 102 females and 87 males on average 4.21 ± 2.73 years old. Approximately 49% (92/189) of the dogs used a deltamethrin collar (repellent collar). The genetic relationship matrix of the first two principal components (C1 and C2) demonstrated that the sample structure was characterized by the grouping of the individuals into 4 breed-related clusters (one homogeneous and three heterogeneous clusters) dominated by three breeds (Fig 1). Rottweilers clustered in a homogeneous set with a tendency to the principal component C2. Labrador Retrievers clustered with Italian Greyhounds and Cocker Spaniels in a set prone to the principal component C1, and German Shepherds clustered along with Belgian Shepherds and White Swiss Shepherds in a set intermediate between C1 and C2. Golden Retrievers grouped with the multi-breed subset central cluster. Following evaluation of the sample structure, we decided to correct the stratification of the sample using the identity for state matrix (IBS) included in efficient mixed-model association expedited (EMMAX).

### Serological and parasitological diagnosis

Quantitative evaluation of anti-*Lu*. *longipalpis* saliva IgG required the definition of clinical groups that represented the natural conditions of the dogs sampled. Therefore, we initially investigated the frequency of dogs that tested positive for serological and parasitological diagnoses to allow clinical classification according to the adapted clinical staging of CanL. By PCR analysis, 50%, 34.5% and 29% of the tested dogs were positive when lymph node (LN), buccal swab (BS) or conjunctival swab (CS) samples were tested, respectively. The *L*. *infantum* cDNA was amplified in at least one of the tested samples (LN, BS, and CS) of 132 of the 189 dogs. Therefore, the prevalence of infected dogs was approximately 70%, and the swab samples added an increase of 20% of positivity to the LN samples. Regarding the ELISA test, 93 of the 189 dogs had detectable levels of anti-*L*. *infantum* IgG, and therefore, the prevalence detected by ELISA was approximately 49%. When we considered PCR as the gold standard, the ELISA test showed a sensitivity of 53% and a specificity of 50%.

### Clinical response

When dogs were ranked based on staging, adapted from Paltrinieri et al. [17], we found the following results: Group I—21 (11%) dogs were uninfected; Group II—12 (6%) dogs were exposed; Group III—56 (30%) were infected; Group IV—52 (27%) dogs were diseased; and Group V—24 (13%) were severely diseased. From the 189 evaluated dogs, 24 (13%) had clinical parameters and a diagnosis outside the predetermined classification criteria. Therefore, 165 dogs were grouped according to their clinical classification (**Table 1**).

### Variation in anti-SGH IgG response was preserved after correction of the covariate effects

**Fig 2:**
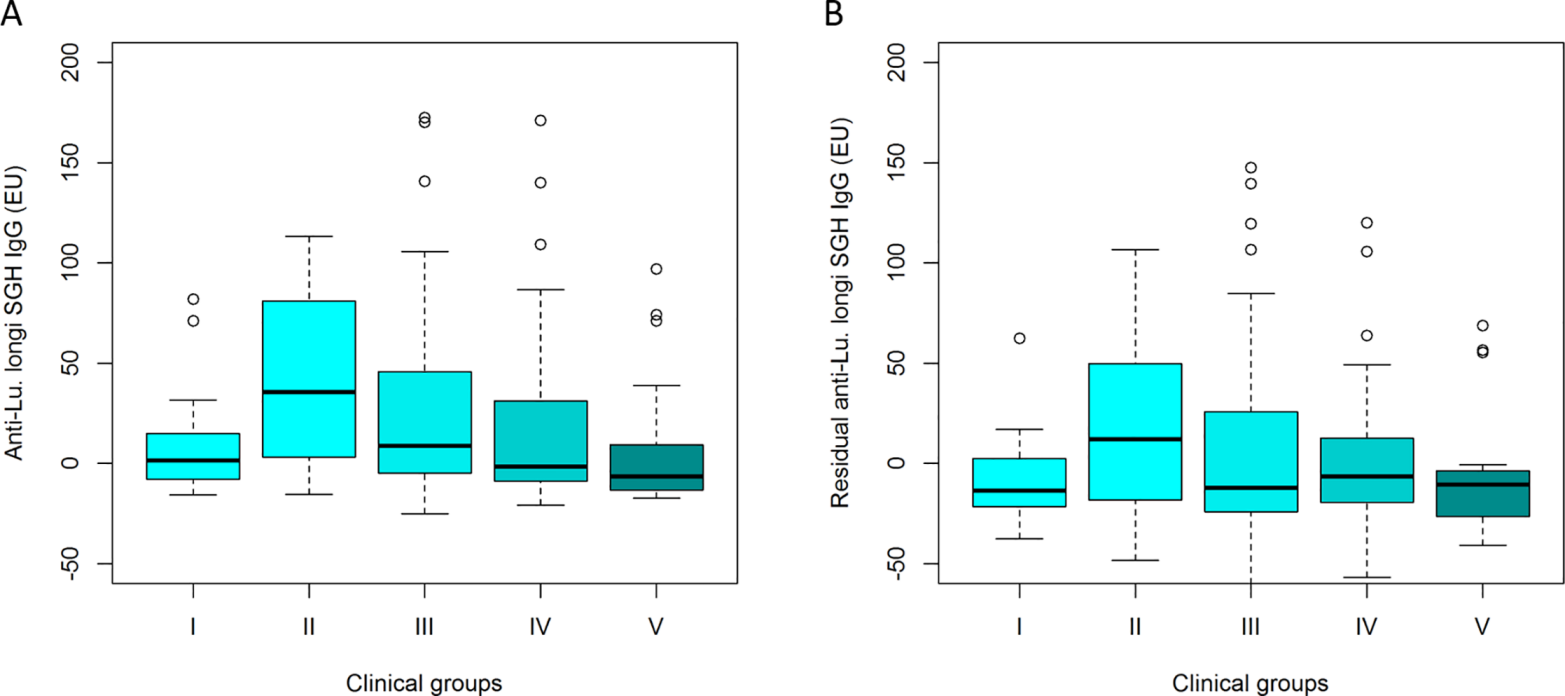
Anti-*Lu*. *longipalpis* SGH IgG levels (ELISA Units) in the sera of dogs relative to the clinical response. IgG levels were measured by ELISA. I—uninfected dogs, II—exposed dogs, III—infected dogs, IV—diseased dogs and V—severely diseased dogs. Anti–SGH IgG levels decreased with the severity of canine leishmaniasis before (A) and after (B) adjustment in the linear regression model (residual). The phenotypic variation of anti-SGH IgG levels was maintained after adjustment.

The levels of anti-*Lu*. *longipalpis* SGH IgG were measured in the sera of dogs from endemic areas to detect if the variation in the anti-SGH IgG response among clinical groups was maintained after the correction for the effect of the fixed-effect variables in the linear regression model. Anti-*Lu*. *longipalpis* SGH IgG levels were higher (p ≤ 0.05) in exposed (II) and infected (III) dogs than in severely diseased dogs (V) (**Fig 2A**). These data support the hypothesis that anti-sand fly saliva antibody contributes to host protection. Alternatively these data could also indicate that diseased dogs were exposed to infected sand fly bites earlier than to not infected sand flies and, hence, became infected. Among the fixed-effect covariates, only the geographical origin of the dogs had a significant association with the anti-*Lu*. *longipalpis* SGH Ig levels (p = 0.002), while age (p = 0.264), sex (p = 0.352) and use of a repellent collar (p = 0.502) were not significantly associated in the analysis of the adjusted mixed linear model. The variation of anti-*L*. *infantum* IgG levels among clinical groups was also preserved even after correcting for fixed-effect covariates in the mixed linear regression model residual (**Fig 2B**).

### Genome-wide analysis of the anti-*Lu*. *longipalpis* SGH IgG response

**Fig 3:**
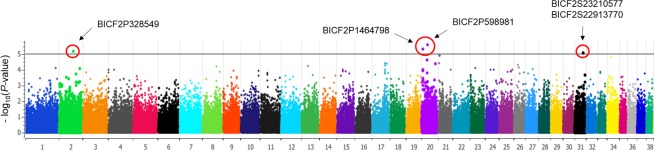
Genome-wide analysis identified 5 markers associated with the anti-*Lu*. *longipalpis* SGH IgG levels (p < 10^−5^) on chromosomes 2, 20 and 31.

**Fig 4:**
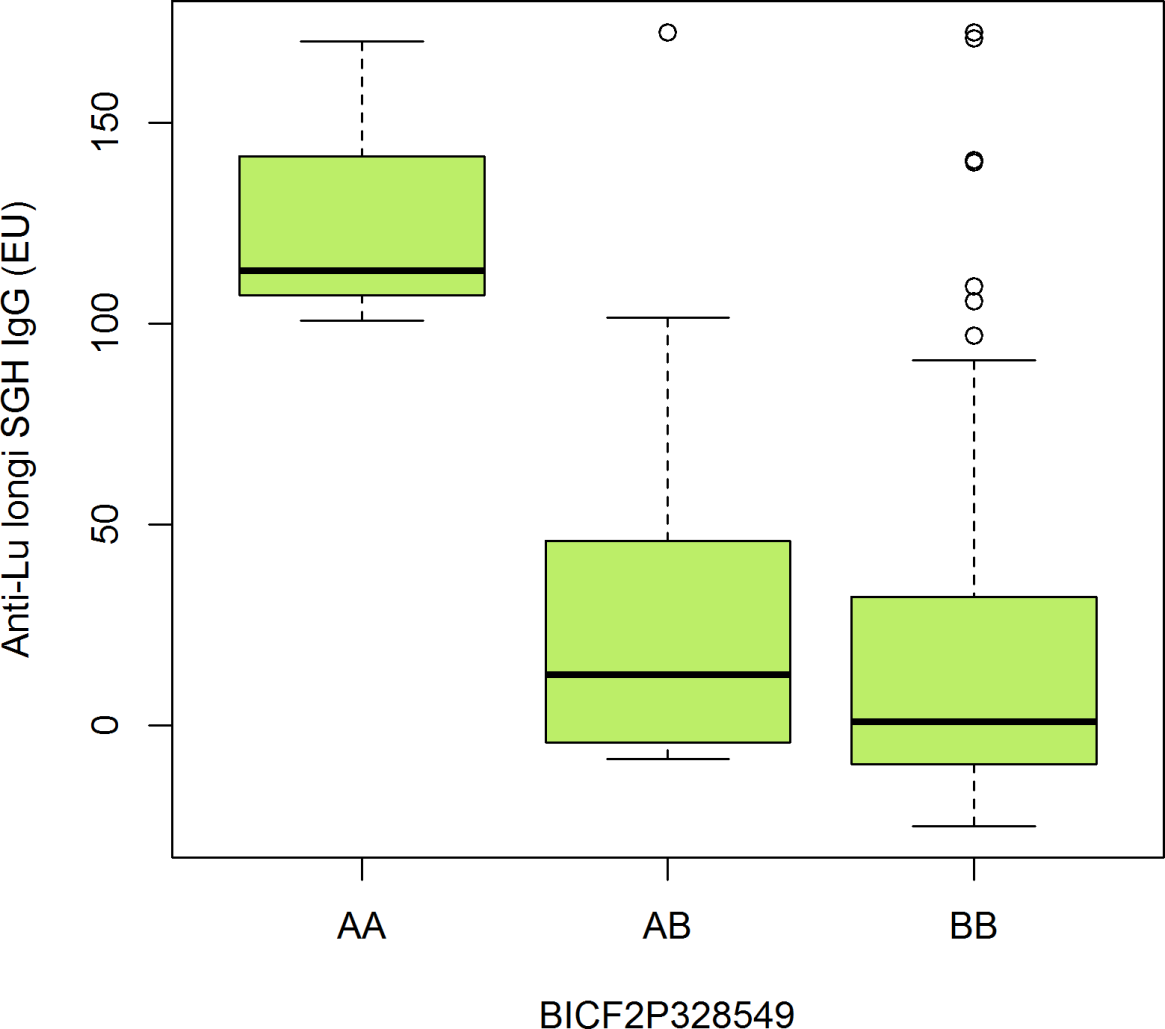
Comparison of anti-*Lu*. *longipalpis* SGH IgG levels among the genotypes of the SNP BICF2P328549, close to positional candidate *CD180*. Higher anti-*Lu*. *longipalpis* SGH IgG levels in allele A carriers (A/B Illumina code).

Analysis for anti-*Lu*. *longipalpis* SGH IgG was conducted with 181 dogs and 109,882 SNPs upon filtering. The anti-*Lu*. *longipalpis* SGH IgG variance explained by genome-wide markers was estimated to be approximately 45%, and the inflation factor (pseudo-lambda) was 0.99, demonstrating control of the polygenic and sample structure effects. Five markers mapping to 4 distinct *loci* were genome-wide significant (**Fig 3**). The significant SNP (BICF2P328549, p = 6.5 x 10^−6^) mapped to position 2:52,946,782, approximately 286 kbp from *CD180* (**Table 2**). This gene encodes a molecule (CD180 or RP105), which, along with MD-1, forms a cell surface receptor belonging to the family of the toll-like receptor. Homozygous carriers of the A allele showed high serum levels of anti-*Lu*. *longipalpis* SGH IgG (**Fig 4**). On chromosome 20, two markers were significant. The SNPs BICF2P1464798, p = 4.86 × 10^−6^, and BICF2P598981, p = 2.5X10^-6^, mapped on 49,31,871 and 22,812,312, respectively (**Table 2**). This region comprises genes such as *RAF1* and *MITF*. The protein encoded by *RAF1* (Raf1) is a serine/threonine-protein kinase that regulates ERK activation in B lymphocytes. Microphthalmia-associated transcription factor (Mitf) is a transcription factor that regulates B-cell differentiation into antibody-secreting plasma cells. The other two significant markers, BICF2S23210577 and BICF2S22913770, 8.39x10^-6^, were mapped in the same region on chromosome 31, which harbors the genes *DIRK1A* and *SIM2*, reported as being related to proliferation and regulation of antimicrobial peptide secretion, respectively [20,21] (**Table 2**).

**Table 2:** Position, frequency, and significance of significantly associated SNPs and positional candidate genes potentially associated with the phenotypes.

Phenotype	SNP	Chromosome position	Frequency	*P*-value	Genes
anti-*Lu*. *longipalpis* SGH IgG	BICF2P328549	2:52946782	7,60%	6.5 × 10^−6^	*CD180*
	BICF2P1464798	20:4931871	43,20%	4.86 × 10^−6^	*RAF1*, *RBSN*
	BICF2P598981	20:22812312	39%	2.5X10^-6^	*MITF*
	BICF2S23210577	31:31558977	37,6%	8.39x10^-6^	*DIRK1A*
	BICF2S22913770	31:31580371	37,6%	8.39x10^-6^	*SIM2*

*CD180* –gene for CD180; *RAF1* –gene for Raf-1 Proto-Oncogene, Serine/Threonine Kinase; *RBSN*–gene for Rabenosyn-5; *MITF*–gene for Microphthalmia-Associated Transcription Factor; *DIRK1A* - Dual Specificity Tyrosine-Phosphorylation Regulated Kinase 1A; *SIM2*—Single-Minded Family BHLH Transcription Factor 2.

## Discussion

The triggering of a specific response against sand fly saliva seems to be related to the host protection. This study employed a genome-wide approach and found *loci* that may improve the understanding of this response. To accomplish this aim, we used EMMAX. This approach has allowed for an increase in the significance of true associations replicated in independent samples and reduces the significance of spurious associations more effectively than other methods that take the sample structure into account [18]. Since EMMAX properly corrects likely polygenic effects and sample stratification in association tests, it was considered a suitable tool in GWASs of complex diseases, which are controlled by polygenes with small effects. Efforts involving EMMAX include discovery of susceptibility *loci* for CanL [19], *loci* associated with canine cell-mediated response to *L*. *infantum* [22] and susceptibility human VL *loci* replicated in two independent samples [23]. In fact, the combination of an adjusted linear regression model with EMMAX restricted genomic inflation to acceptable levels even in a sub-structured multi-racial panel as observed in the present study.

Although they oppose the profile of immunity observed against recombinant *Lu*. *longipalpis* salivary proteins [24], the phenotypic data in the present study indicate that the antibody response against total *Lu*. *longipalpis* SGH was more intense in exposed and subclinically infected dogs than in dogs with severe CanL. The group of exposed dogs previously described [17] consists of dogs living in endemic areas with a weak anti-*Leishmania* antibody response; however, there were no detectable parasites in the tissues. The highest levels of anti-*Lu*. *longipalpis* SGH IgG reinforce the idea of exposure of these dogs to infected sandflies and points to a potential role of anti-*Lu*. *longipalpis* SGH antibodies, which has recently been shown to be consistent in avoiding the manifestation of clinical signs in the long term [25]. The absence of infection or clinical signs in dogs with the higher levels of anti-*Lu*. *longipalpis* SGH IgG supports the hypothesis that these antibodies facilitate triggering of an effective cell-mediated response against *L*. *infantum*. Indeed, response as a delayed hypersensitivity (DTH) at the bite site in the skin of dogs previously exposed to *Lu*. *longipalpis* has been demonstrated [7], as has the anti-*Leishmania* DTH more frequently observed in humans with high levels of anti-*Lu*. *longipalpis* saliva IgG [8]. The mechanisms by which the anti-saliva antibody response could contribute for a cell-mediated response and protection in the natural pre-exposure to the vector remains unknown. However, experimental efforts suggest that the anti-saliva antibodies could neutralize the synergistic effects of SGH with the *L*. *infantum* infection that include increased apoptosis, reduced reactive oxygen species (ROS) production, and increased prostaglandin E2 (PGE2) production by neutrophils, which promotes macrophage recruitment and inhibition of proinflammatory cytokines [26, 27, 9]. Thus, anti-*Lu*. *longipalpis* SGH IgG could contribute to the control of infection in the early steps [7]. The possibility of antibodies increasing the uptake of saliva antigens by antigen presenting cells to generate cell-mediated immunity requires further investigation.

The absence of significant association between the use of repellent collar and anti- *Lu*. *longipalpis* antibody response may indicate a low coverage of the use of the repellent collar in the endemic areas evaluated in the present study. Sevá et al. [28] demonstrated the blockade of 100% transmission of *L*. *infantum* for dogs and humans when the use of the collar covered 90% of the canine population. Furthermore, the absence of the association may reflect the adoption of the collar only after the diagnosis of *L*. *infantum* infection, in an attempt to block the transmission of parasite to the family members.

Anti-SGH antibodies levels were lower in dogs that were exposed long enough to express the disease. This data indicates that the anti-SGH antibody response is not an infallible indicator of exposure to the sandfly and that other factors, such as the genetic basis, may influence the anti-SGH antibody response. Candidate genes mapped in regions associated with the quantitative data of anti-*Lu*. *longipalpis* SGH IgG are related to the variation in the ability to trigger a rapid antibody response. For example, *CD180* (also known as *RP105*) is an orphan member of the toll-like receptors (TLR4) family that triggers BCR-like B lymphocyte activation and rapid polyclonal IgG production regardless of the mediators of toll signaling [29, 30, 31]. Chaplin et al. [31] showed that targeting antigen (Ag) to CD180 induced a strong Ag-specific IgG response, immunological memory and affinity maturation mainly dependent on B cells expressing both CD180 and Ag-specific BCR. In fact, the authors also noted that IL-4, IFN-α and mature B cells are not required for Ag targeting to the CD180 IgG response, suggesting that T1 B cells expressing CD180 and specific BCR could quickly mature into plasma cells secreting IgG. Additional studies will be needed to assess whether the segment identified in chromosome 2 may indicate variations in CD180 expression that determine the variation in the precocity of the anti-*Lu*. *longipalpis* SGH IgG response.

The product of another positional candidate gene, the transcription factor Mitf, was highly expressed on naïve B-lymphocytes, where it inhibited differentiation into antibody-secreting plasma cells, antibody and autoantibody secretion by inhibiting IRF-4 [32]. A mutation in a melanocyte-specific *MITF* gene promoter was related to the white coat color in two breeds of dogs [33]. Therefore, the white coat coloration in a multi-breed panel could represent a possible confounding factor for the association between genotypes and anti-*Lu*. *longipalpis* SGH IgG levels. However, we performed a case-control GWAS between dogs of our panel belonging to breeds characterized by white spots on the coat, in accordance with the study of Karlsson et al. [33]. In this additional GWAS, we observed that regions with significant signals for white coat color differed completely from regions with significant associations in GWASs for anti-*Lu*. *longipalpis* SGH IgG, suggesting the existence of pleiotropic immunological and coat color roles for the *MITF* locus.

The protein Raf acts as a regulatory link between membrane-associated Ras GTPase and the MAPK/ERK cascade involved in cell differentiation and proliferation. This protein is involved in the activation of B cells via BCR [34]. Similar to *CD180*, these genes are related to B cell activation and production of antibodies. Therefore, the identified markers point to a region that could be the object of studies that aim to identify variations in the ability to secrete anti-*Lu*. *longipalpis* SGH IgG.

Given the increasing interest in the use of the components of saliva of *Lu*. *longipalpis* in the protection against *L*. *infantum* [8, 7, 35], the genomic regions identified in the present study are noteworthy for studies aimed at confirming the role of proteins encoded by the candidates genes for the anti-*Lu*. *longipalpis* SGH response. Therefore, our findings open a new perspective for the discovery of adjuvants for vaccines containing components of sand fly saliva in prophylactic strategies against visceral leishmaniasis.

## Supporting information

S1 File Supporting phenotype and genotype data. (ZIP)
